# Toothed Whales Have Black Neurons in the Blue Spot

**DOI:** 10.3390/vetsci9100525

**Published:** 2022-09-26

**Authors:** Simona Sacchini, Antonio Fernández, Blanca Mompeó, Raquel Ramírez, Manuel Arbelo, Unn Holgersen, Oscar Quesada-Canales, Ayoze Castro-Alonso, Marisa Andrada

**Affiliations:** 1 Veterinary Histology and Pathology, Veterinary School, Institute of Animal Health, University of Las Palmas de Gran Canaria, c/Transmontaña s/n, 35416 Arucas, Spain; 2 Department of Morphology, Campus Universitario de San Cristobal, University of Las Palmas de Gran Canaria, c/Blas Cabrera Felipe s/n, 35016 Las Palmas de Gran Canaria, Spain; 3 Nordland Research Institute, P.O. Box 1490, 8049 Bodø, Norway

**Keywords:** neuromelanin, neurodegeneration, locus ceruleus, cetaceans, beaked whales, dolphins, aging

## Abstract

**Simple Summary:**

Neuromelanin is a dark pigment that is present in several types of neurons of the brain. The role of human neuromelanin is a matter of controversy and, over the past few years, has been attributed to having a dual nature, either in a protective role to shield neurons from toxic compounds, or as a trigger of neuroinflammation. This pigment has been researched mainly in the human brain, but it has also been found in the neurons of monkeys, horses, giraffes, cattle, sheep, goats, dogs, rats, and even in frogs and tadpoles. Even so, neuromelanin in humans and primates presents unique features that are not shown in other animals. A study on the morphology of the locus ceruleus (a key brain structure) of the family *Delphinidae* highlighted the presence of a large amount of neuromelanin within this brain area. In an attempt to better define the ultrastructure of neuromelanin in toothed whales, two brain specimens of the suborder Odontoceti were investigated. The two toothed whales that were examined presented melanin granules associated with lipid droplets and membranes that bore a striking resemblance with human neuromelanin. Its accumulation takes place over the entire life span, and appears to contain the story of one’s life exposure to several endogenous and environmental metals and/or compounds.

**Abstract:**

Neuromelanin (NM) is a dark polymer pigment that is located mostly in the human substantia nigra, and in the locus ceruleus, referred to as “the blue spot”. NM increases linearly with age, and has been described mainly in the human brain; however, it also occurs in the neurons of monkeys, horses, giraffes, cattle, sheep, goats, dogs, rats, and even in frogs. While in most of these mammals NM shows the histochemical and ultrastructural features typical of lipofuscins, human NM is confined within cytoplasmic organelles that are surrounded by a double membrane, suggesting an autophagic origin. In a study on the morphology of the locus ceruleus of the family *Delphinidae*, the presence of a variable quantity of NM in the interior of locus ceruleus neurons was observed for the first time; meanwhile, nothing is known about its ultrastructure and composition. Transmission electron microscopy demonstrated in two toothed whales—an Atlantic spotted dolphin (*Stenella frontalis*; family *Delphinidae*) and a Blainville’s beaked whale (*Mesoplodon densirostris*; family *Ziphiidae*)—the presence of melanin granules associated with lipid droplets and membranes that were very similar to that of human NM. The relationship between NM and neuronal vulnerability must be studied in depth, and cetaceans may offer a new natural-spontaneous comparative model for the study of NM and its implication in neurodegenerative diseases.

## 1. Introduction

Neuromelanin (NM) is a dark polymer pigment that is particularly concentrated in several groups of neurons in the brain. It increases linearly with age [1] in the dopaminergic neurons of the substantia nigra (SN) and in the noradrenergic neurons of the nucleus ceruleus [caeruleus] [2] (LC), also known as the “the blue spot”. NM can be localized in the ventral portion of the neuraxis, where the presence of NM-containing neurons is proportional to their chemical and metabolic characteristics and functional specialization [3]. NM has been shown to accumulate in other human brain regions during aging, such as the putamen, the premotor cortex, and the cerebellum, partially resembling the NM present in the SN and LC [1]. NM and lipofuscin have been proposed to originate from incompletely degraded proteins and lipids that are principally derived from the breakdown of mitochondria or products of oxidized catecholamines [4]. Furthermore, NM results from peroxidation of the lipofuscin granules [5].

The purpose of human NM is a matter of controversy, and over the past few years it has been given a dual nature, either in a protective role for shielding neurons from toxic compounds, or as a trigger of neuroinflammation, activating neuroglia when NM is released by dying neurons. NM seems to continuously accumulate metals throughout aging without turnover, serving as the most effective system for scavenging and for long-term immobilization of toxic metals that invade neurons [1]. On the other side, it has been proposed that pigmented neurons are more vulnerable than non-pigmented neurons [6]. In fact, there is a significant loss of LC NM-containing neurons in individuals with Alzheimer’s (AD) and Parkinson’s (PD) diseases [7,8,9].

NM has been described mainly in the human brain, but also in the neurons of monkeys, horses, giraffes, cattle, sheep, goats, dogs, rats, and even in frogs and tadpoles (*Rana esculenta*) [3,10,11,12,13]. Even so, NM in humans and primates presents unique features that are not found in other animals. Indeed, human NM ultrastructure can be observed as an electron-dense melanin polymer, a component of intermediate electron density, with an electron-lucent lipid constituent [14].

Cetaceans are homeotherms, long-lived species, and are located at the top of the marine food chain. Thus, they are considered to be bioindicator species and sentinels of the health of the sea. Recent studies have latterly presented some evidence that cetaceans may be one of the very few comparative natural models for AD, as well as for other neurodegenerative diseases (NDDs) [15,16,17,18,19,20,21,22,23]. The lifespan of many species of these animals approximates that of humans. This makes cetaceans, particularly odontocetes, a new and more authentic comparative natural model for the study of certain NDDs in humans. A study on the morphology of the LC of the family *Delphinidae* highlighted the existence of a large amount of NM within the LC neurons (LC-NM) [18]. NM had not been previously observed in any cetacean’s catecholaminergic area of the brain [24,25,26,27]. Most neurons, although not all of them, exhibited typically dark brown to blackish NM granules within the cytoplasm. NM granules occupied either a pole of the cell, or even half of the cytoplasm [18]. This fact represents a striking difference with the situation of the closely related terrestrial *Cetardiodactyla* [10], which show no NM granules in the SN and the LC. In an attempt to better define the LC-NM ultrastructure of toothed whales, two specimens of the suborder Odontoceti, an Atlantic spotted dolphin (*Stenella frontalis*; Cuvier, 1829; family *Delphinidae*) and a Blainville’s beaked whale (*Mesoplodon densirostris;* De Blainville, 1817; family *Ziphiidae*), were investigated.

## 2. Materials and Methods

Systematic pathological studies were performed on the carcasses, in order to determine the cause of death and/or stranding. Required permission for the management of stranded cetaceans was issued by the environmental department of the Canary Islands’ Government, and by the Spanish Ministry of Environment. Different age categories were used: fetus-neonate-calf, juvenile-subadult, adult, and elderly. The categories were established on the basis of total body length, gross and microscopic gonadal appearance [28], and systemic gross and microscopic features, e.g., pronounced tooth wear, neuronal lipofuscinosis, amount of neuromelanin in the LC or SN, intraneuronal polyglucosan bodies, leptomeningeal fibrosis, and choroid plexus hyalinosis.

Two brain specimens of the suborder Odontoceti were used for this study. One brain was from an adult male Atlantic spotted dolphin (ASD), and the other was from an adult female Blainville’s beaked whale (BBW), both stranded on the Canary Islands in 2016 and 2017, respectively. Additional data are shown in Table 1.

The brains were removed and immersion-fixed at the time of necropsy in 10% neutral-buffered formalin (4% formaldehyde solution, pH 7.4). The two brains were processed for histology and histopathology, according to the methodology implemented in our laboratories [30].

**Figure 1:**
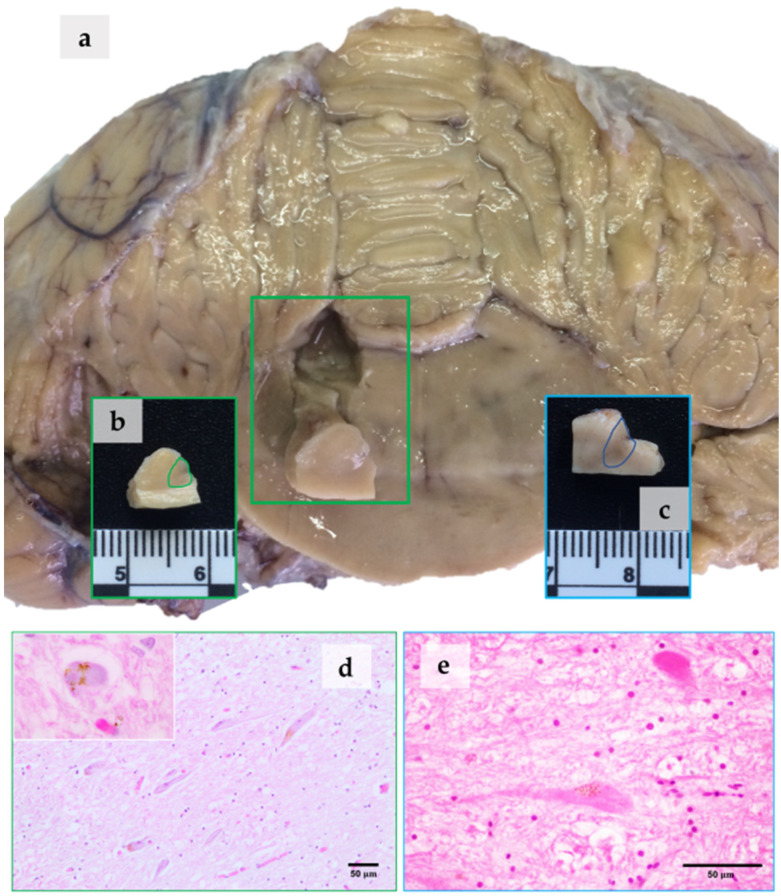
Sampling of the LC and presence of NM. ASD, blue boxes; BBW, green boxes. Samples were taken from the LC at the level of the metencephalon (**a**), and more exactly from the A6d and A6v subdivisions ((**b**,**c**), marked areas). In the ASD, the LC can be seen macroscopically as a darkened area ((**c**), marked area). Neuromelanin in LC perikaryal cytoplasm, observed as light brown granular material. Hematoxylin and eosin staining (**d**,**e**). Inset: round pale basophilic inclusions in LC neuronal perikaryon, pushing NM to the periphery. BBW (**d**).

The remaining part of the study was carried out at the University of Cordoba (Spain). A small sample of the LC (Figure 1a), proceeding from the A6d and A6v subdivisions (Figure 1b,c, marked areas), and no thicker than 5 mm, was taken for ultrastructural studies. LC samples were fixed in 2% glutaraldehyde in 0.1 M sodium cacodilate buffer (pH 7.4), at 4 °C for 12 h. The samples were postfixed in 1% osmium tetroxide in 0.2% veronal buffer (pH 7.4) for 30 min, according to Sabatini [31]. After that, the tissues were rinsed in phosphate-buffered saline (PBS; pH 7.4), gradually dehydrated in an alcohol series, and embedded in Araldite. Thin sections were stained with uranyl acetate and lead citrate. A JEOL JEM-1400 (JEOL Ltd., Tokyo, Japan) transmission electron microscope was used to investigate the existence of organelles containing melanic pigments in the neurons of the LC.

## 3. Results

### 3.1. NM in the LC

To the naked eye, the LC samples from the two animals appeared to be different (Figure 1a–c). The sample from the ASD (Figure 1c) was darker than that from the BBW (Figure 1b). When observed using an optical microscope, the neurons of the LC in both animals were characterized by the presence of NM granules that were located in the neuronal perikaryon (Figure 1d,e). In hematoxylin and eosin sections, brownish granules occupied either a pole of the cell or most of the cytoplasm. In the BBW, round pale basophilic cytoplasmic inclusions surrounded by a rim of NM granules were also observed in hematoxylin and eosin (Figure 1d, inset).

### 3.2. NM Ultrastructure

**Figure 2:**
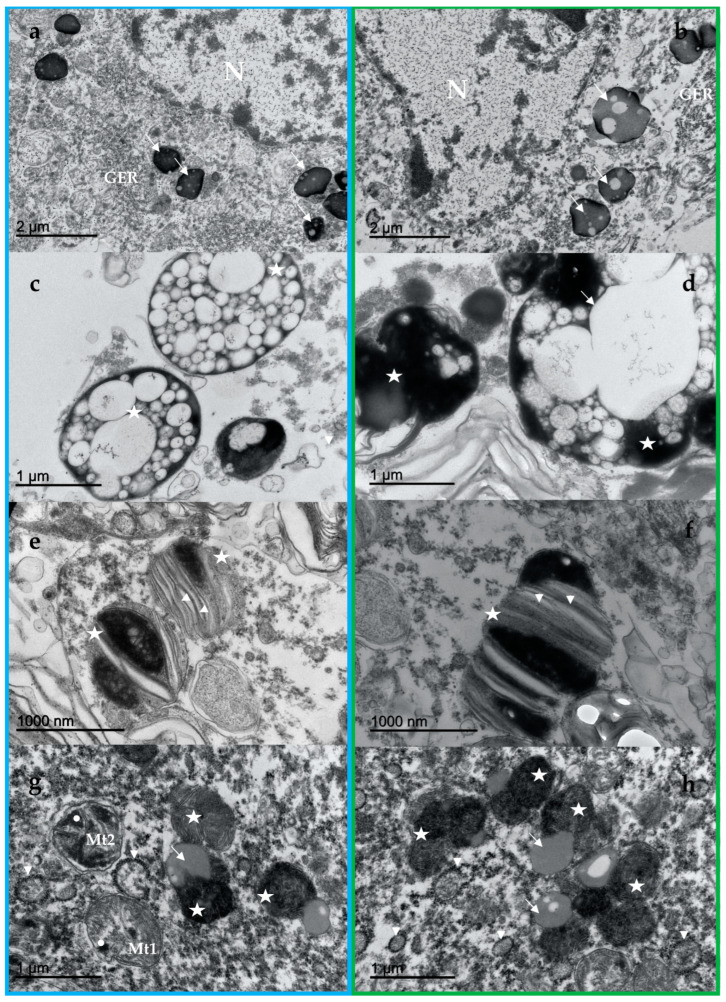
Ultrastructural analysis. ASD, blue box; BBW, green box. Ultrastructure of the neuromelanin via EM (**a**–**f**). Irregular round homogeneous electron-dense melanin granules distributed in dense granules (arrows) throughout the cytoplasm, in this case near the nucleus (N) and the granular endoplasmic reticulum (GER) (**a**,**b**). Numerous variable-sized electron-lucent lipids within electron-dense melanin granules ((**c**,**d**), stars). Lipid droplets fused into a larger one ((**d**), arrow). Melanin granules associated with parallel layers (arrowheads) of membraniform structures ((**e**,**f**), stars). Ultrastructure of the lipofuscin via EM (**g**,**h**). Lipofuscins present a “fingerprint-like” profile (stars) with round inclusions (arrows)). A preserved/normal mitochondria (Mt1 (**g**)) and a degenerating mitochondria (Mt2, (**g**)) with dense granules in their matrix (white circles), and polysomes ((**g**,**h**), arrowheads) are also present.

In the ultrastructural study, the two examined animals principally showed three types of neuronal pigment granules: (1) electron-dense and homogeneous melanin granules (Figure 2a,b, arrows); (2) electron-dense melanin granules associated with electron-lucent lipids (Figure 2c,d, stars); and (3) melanin granules associated with membranes (Figure 2e,f, stars). The three types of granules were present in both animals, differing fundamentally in their size and quantity.

Highly electron-dense matter was present in organelles up to 3 μm in size, and either occurred in clumps or were separate (Figure 2a,b, arrows). The melanin granules associated with lipid droplets were present as melanin that was distributed in dense granules throughout the cytoplasm (Figure 2c,d). The lipid droplets were initially observed as small vacuoles (Figure 2c, stars), but when the granule increased appreciably in size, they resembled fatty droplets blackened by melanin (Figure 2d, arrow). In the BBW, these particular granules were more numerous and larger in size (Figure 2d).

The melanin granules that were oriented in sheets were very abundant in the ASD, located alone or in accumulations (Figure 2e). The formations showed a body of melanin that was associated with membraniform structures, in layers and parallel to each other (Figure 2e,f, arrowheads). These granules were small in size, and were transforming and presenting as membranous, large-sized structures. In the BBW, the melanin granules that were oriented in sheets were abundant, clumping and generating larger granules (Figure 2f).

### 3.3. Lipofuscin Ultrastructure

Clumps of lipofuscins belonging to the lipoxin complex, with irregular and pleomorphic bodies, were also observed. Lipofuscins presented a “fingerprint-like” profile (Figure 2g,h, stars), with homogeneous round inclusions of variable size (arrows).

### 3.4. Other Elements of the LC Neurons

Neurons of the LC displayed a knight cytoplasm and nucleoplasm with a clearly defined heterochromatin pattern, as well as intact organelles. Nissl bodies consisted of a number of granular endoplasmic reticula (Figure 2a,b, GER) whose membranes were covered by ribosomes, and free polysomes (Figure 2g,h, arrowheads) composed of a great number of ribosomes occurred in the cytoplasmic matrix (30–40). The agranular reticulum consisted of cisternae and tubules that were devoid of membrane-bound ribosomes. The Golgi apparatus was also evident as a stack of curved cisternae that lacked attached or free ribosomes. Glycogen particles were also evident (not shown). Well preserved mitochondria (Figure 2g, Mt1) as well as degenerating mitochondria (Figure 2g, Mt2), as well as some mitochondrial remains, were also present in both animals. Some mitochondria showed dense granules in their matrix (white circles).

## 4. Discussion

### 4.1. NM at the Optic Microscope

It is important to highlight that in routinely prepared formalin-fixed paraffin-embedded hematoxylin and eosin sections, NM is visualized as a brownish pigment. This can easily lead the observer to mistakenly identify NM as lipofuscin. On the other hand, the use of cryosectioning and Nissl staining (as thionine staining) more clearly show the real dark brown-blackish appearance of the NM granules, as observed in our previous research on LC morphology in the family *Delphinidae* [18]. This ultrastructural study finally confirmed the identification of the NM granules correctly.

### 4.2. NM Ultrastructure and Its Implications

The two examined toothed whales presented melanin granules that were associated with lipid droplets and membranes that bore a striking resemblance with human NM. The size of the NM granules (up to 3 μm) was very similar to the granule size observed in humans and rhesus monkeys (*Macaca mulatta*), up to 2.5 μm [32]. We portrayed three main constituents: (1) electron-dense and homogeneous melanin granules; (2) electron-dense melanin granules associated with electron-lucent lipids; and (3) melanin granules associated with membranes. The three types of granules were present in both animals, differing fundamentally in their size and quantity.

Moses et al. also described three types of components in LC and SN-NM granules, both in the human and rhesus monkey. The first component is a finely granular, medium density matrix which occasionally has linear configurations that are very similar to those of neuronal lipofuscin granules. The second component is a very dense, coarsely granular material which appears to be deposited on the finely granular matrix, and is apparently the reducing part of the NM granule. The third component of the NM granule is a lipid globule [32]. Likewise, human NM has been described as complex bodies that include a very dense component, and vacuole-like structures [33]. Membrane-bound lipid bodies were observed in the two analyzed animals, and their major composition in the NM from human SN is dolichols, with lower amounts of other lipids [34]. In the human brain, NM organelles are membrane delimited and, in some cases, also present a double membrane, as in the case of mitochondria; these features identify the NM organelles as autophagic vacuoles [4,8]. In fact, NM is insoluble, and cannot be degraded by lysosomes; thus, it remains trapped within undegraded or partly degraded autophagic structures [4]. Protein analyses revealed that lysosomal proteins are the major class of proteins in NM-containing organelles [34]. NM seems to be a byproduct of a macroautophagic pathway in which autophagic vacuoles engulf intracellular components to later fuse with lysosomes, in order to degrade their constituents, thus accumulating over a lifetime [4].

Finally, in relation to other neuronal components, the neuronal Nissl structure and abundance are directly correlated with the rate of protein synthesis that occurs there [35]. On the other hand, the dense granules that are observed in the matrix of mitochondria are usually associated with mitochondrial aging [36].

### 4.3. NM Ultrastructure in Other Animals and Age-Dependent Accumulation

In analyzing the NM ultrastructure in other species, it is noteworthy that horse NM is more similar to lipofuscin, with a typical lamellar pattern [11]. Moreover, in ruminants (cattle, sheep, and goat), NM shows the histochemical and ultrastructural features typical of lipofuscins [10]. In the frog and tadpole, it is difficult to discern whether the observed pigment is really NM or not, as it is detected in early stages of development of the nervous system (tadpole) [3,12]. The presence of NM pigment in neurons of the rat SN is a matter of controversy, with only 5% of nigral neurons present NM [37].

Dogs at 1 year of age exhibit a similar situation, while the SN from adult and aged dogs displays abundant and larger NM-coalesced granules that form irregular bodies containing several lipid globules, with enough dense material to hide the underlying matrix [37].

As a matter of fact, Brayda describes three stages in the progression of NM in the dog SN [38]:

- In the first stage, there is no pigmentation visible by optical microscope; there are a few lysosome-like organelles of medium electron density, surrounded by a single membrane, 0.5 to 1 μm in size, and occasionally lipidic globules and rarely fingerprint-like structures (dogs less than 1 year).
- In the second stage, highly electron-dense organelles, 1.5 to 3 μm in size, are surrounded by an irregular membrane. The lipidic component is more abundant, as well as the electron-dense granular matrix. Optically, only a few small NM granules are visible (dogs up to 5 years)
- In the third stage, NM shows great polymorphism, and contains a larger quantity of highly electron-dense organelles, 2 to 4 μm in size, and lipid droplets. At this stage, groups of organelles merge and form larger and more numerous electron-dense masses. Optically, NM granules are quite visible (dogs aged 6 years onwards).

The accurate age of the two adult cetaceans, a male ASD and a female BBW, were unknown, as they were wild animals; nevertheless, age could determine the size of the granules, as seen in dogs. As a matter of fact, the NM granules were larger in the BBW than in the ASD, leading us to believe that the BBW was older than the ASD.

Even if this was not the goal of our research, we can assume that age-dependent NM accumulation studies and typification may help to establish a novel method to determine age in these elusive wild animals. Future large-scale studies may facilitate the establishment of new parameters, namely NM quantity, size, and type, which could confirm the approximate ages of the animals, and furthermore predict related NDDs.

In fact, the development of the first rodent model that produced human-like NM (hTyr-overexpressing rodents) in dopaminergic SN neurons has made it possible for the first time to experimentally assess the consequences of age-dependent NM accumulation up to levels reached in elderly humans [39,40]. However, other animals, especially toothed whales, may offer new insights in our understanding of NM and its association with NDDs in general, and with PD in particular.

### 4.4. NM and Neuronal Vulnerability

Our findings bring attention back to the selective vulnerability of specific neuronal groups [41]—in this case, neurons containing NM. As such, NM-containing neurons in different brain regions have been found to consistently degenerate in PD. These include the noradrenergic neurons of the LC, whose degeneration leads to characteristic non-motor symptoms [42]. In fact, severe lesions of the neurons have been described in the A6 division of the LC. The LC innervates the entire forebrain, and A6 Parkinson’s dementia correlates with a marked loss of forebrain Ch4 cholinergic neurons in the nucleus basalis [42]. In vivo MRI studies have also shown age-related declines in NM-sensitive cells in the human LC, with a greater reduction in males than in females [43,44]. NM may represent a cellular mechanism that evolved within catecholaminergic neurons to protect these cells from the potentially toxic metabolic products of these neurotransmitters [45]. By contrast, the PD etiopathogenesis appears indissolubly linked to the presence of NM. In fact, the intracellular stockpiling of NM jeopardizes neuronal function when it accumulates above a specific threshold. The outcome is the triggering of PD, including Lewy body inclusions [40].

While it is not the purpose of our study to advance pathological hypotheses, from a visual standpoint the BBW presented a lighter aspect in the LC, thus suggesting a loss of NM. Undertaking future large-scale studies may contribute to the understanding of the mechanisms underlying selective neuronal vulnerability in toothed whales.

### 4.5. NM-Related Intraneuronal Inclusions

It was not possible to establish the identity of the round inclusions that were found in the LC of the BBW (Figure 1d, inset). Extrasomal inclusions were also observed (not shown), but it was not possible to establish their exact nature (Lewy, pale, Marinesco or Lafora bodies, among others). Lewy bodies and their precursor, pale bodies, were observed in NM-containing neurons and related to Lewy pathology. The Lewy pathology consists of the protein, α-synuclein, in an insoluble form. Alpha-synuclein redistributes to NM pigment in the early stages of PD, and becomes trapped within NM granules [46]. Then, α-synuclein pathology becomes widespread in the central and peripheral nervous system [47]. Alpha-synuclein- and ubiquitin-positive inclusions have been already found in the mesencephalon of a deep diver toothed whale, a short-finned pilot whale (*Globicephala macrorhynchus*) [30]. It has been shown that neuronal MHC-I is also induced by factors released from microglia which are activated by NM or α-synuclein, substances found extracellularly in postmortem PD brains [48]. Hence, NM and associated inclusions may contribute to making cetaceans’ brains more defenseless, in particular the LC and SN areas.

In conclusion, NM is an enigmatic pigment, but it is irrefutably not an inert waste product of neuronal metabolism, as previously believed [49]. In fact, NM accumulation takes place over the entire life span of an organism, and the NM pigments appear to contain the story of one’s life exposure to several endogenous and environmental metals [1]. Our study was aimed at elucidating the ultrastructural morphology of LC-NM, and to understand its possible implications. On the other hand, we observed some pathological aspects that would require further large-scale studies in order to decipher their possible correlation with NDDs. Incidentally, the two analyzed toothed whales were also included in a previous study on NDDs from our team [23]. The BBW (case 5) had widespread granulovacuolar cytoplasmic labeling to neurofibrillary tangles antibody in Purkinje neurons. Moreover, the animal presented cerebral nasitremiasis that was confined to the forebrain (unpublished data). The ASD (case 6) had mild, multifocal granular to vacuolar cytoplasmic labeling to the neurofibrillary tangles antibody in scattered Purkinje neurons.

The correlation between our previous findings and the results obtained in this study is purely speculative, and exceeds the purpose of our morphological study.

**Figure 3:**
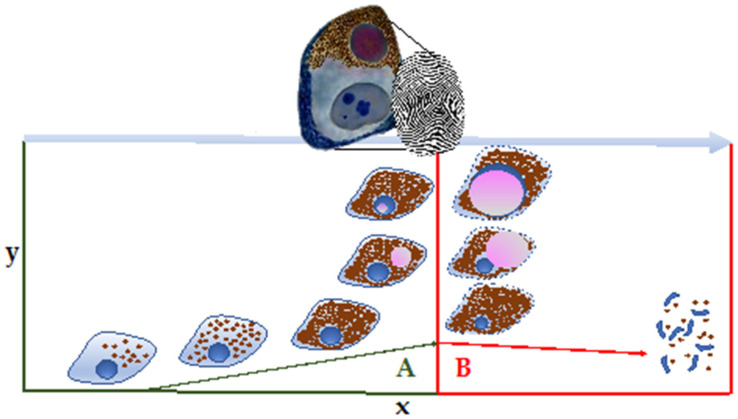
Intranuclear and intracytoplasmic inclusions in NM-containing neurons exhibit a specific fingerprint-like pattern of the real status of some neurons. In “A”, neurons are still healthy, while in “B”, they are proceeding toward death with the activation of microglial (rod) cells and eventual neuronophagia. The activation of microglia by NM can drive antigen presentation by SN and LC neurons, a response that may represent a critical step in PD pathogenesis. Part of the drawing was adapted from Vila, 2019 [39].

Nevertheless, we can assume that NM and its associated revelatory neuropathological inclusions exhibit, in some neurons, a specific fingerprint-like pattern of the history of the brain (Figure 3), and could also help researchers better understand the pathogenesis of some NDDs.

## 5. Conclusions

This is the first study which describes the NM ultrastructure in any marine mammalian brain. This ultrastructural study finally confirmed the authentic identity of NM granules, and demonstrated that NM in toothed whales is morphologically different from lipofuscin, a finding which is very similar in human NM. The significance of NM ultrastructure, composition, and accumulation/depletion in aged animals remains to be elucidated. Chemical and proteomic analyses are also necessary to better investigate and understand the real structure and role of cetacean NM. The mechanism that underpins selective neuronal vulnerability related to NM has yet to be untangled. Our results placed special emphasis on the assumed uniqueness of PD to humans which, in contrast, could be investigated in other mammals such as toothed whales.

## Figures and Tables

**Table 1:** Data of the two analyzed animals. AC, age category; S, stranding; CC, condition code; CD, cause of death. CD determined according to pathologic categories in [29].

Species/Diving Habit	Sex	AC	S	CC	CD
ASD: shallow diver	male	adult	alive	fresh	Interaction with fishing activities.
BBW: deep diver	female	adult	dead	fresh	Pathology associated with significant loss of nutritional status.

## Data Availability

The data presented in this study are available from the corresponding author on reasonable request. Some data may be part of other unpublished studies.
